# Crosstalk between Immune Checkpoint Modulators, Metabolic Reprogramming and Cellular Plasticity in Triple-Negative Breast Cancer

**DOI:** 10.3390/curroncol29100540

**Published:** 2022-09-23

**Authors:** Arpita Poddar, Sushma R. Rao, Prashanth Prithviraj, George Kannourakis, Aparna Jayachandran

**Affiliations:** 1 Fiona Elsey Cancer Research Institute, Ballarat, VIC 3350, Australia; 2 Department of Materials Science and Engineering, Monash University, Melbourne, VIC 3800, Australia; 3 Ian Potter NanoBiosensing Facility, NanoBiotechnology Research Laboratory (NBRL), School of Science, RMIT University, Melbourne, VIC 3000, Australia; 4 Proteomics, Metabolomics and MS-Imaging Facility, South Australian Health and Medical Research Institute, Adelaide, SA 5000, Australia; 5 Adelaide Medical School, University of Adelaide, Adelaide, SA 5005, Australia; 6 School of Science, Psychology and Sports, Federation University Australia, Ballarat, VIC 3350, Australia; 7 Gallipoli Medical Research Institute, Greenslopes Private Hospital, Brisbane, QLD 4120, Australia

**Keywords:** immune checkpoints, EMT, metabolic reprogramming, TNBC, breast cancers, MET, PD-L1

## Abstract

Breast cancer is one of the major causes of mortality in women worldwide. Accounting for 15–20% of all breast cancer diagnoses, the triple-negative breast cancer (TNBC) subtype presents with an aggressive clinical course, heightened metastatic potential and the poorest short-term prognosis. TNBC does not respond to hormonal therapy, only partially responds to radio- and chemotherapy, and has limited targeted therapy options, thus underlining the critical need for better therapeutic treatments. Although immunotherapy based on immune checkpoint inhibition is emerging as a promising treatment option for TNBC patients, activation of cellular plasticity programs such as metabolic reprogramming (MR) and epithelial-to-mesenchymal transition (EMT) causes immunotherapy to fail. In this report, we review the role of MR and EMT in immune checkpoint dysregulation in TNBCs and specifically shed light on development of novel combination treatment modalities for this challenging disease. We highlight the clinical relevance of crosstalk between MR, EMT, and immune checkpoints in TNBCs.

## 1. Introduction

Breast cancer is the most prevalent cancer in women and one of the leading causes of cancer death among women worldwide. It accounts for 12% of all new cancer cases and 25% of all types of cancer in women [1,2]. At the molecular level, based on hormone receptor status, breast cancers are categorized as Luminal A (estrogen receptor (ER)+, progesterone receptor (PR)+, and human epidermal receptor 2 (HER2)-); Luminal B (ER+ and/or PR+, HER2+/−); HER2 enriched (ER-, PR- and HER2+); and Basal-like including triple negative (ER-, PR- and HER2-) breast cancers [3]. Among these subtypes, triple-negative breast cancer (TNBC) accounts for about 15–20% of all breast cancer types, and it is the most aggressive subtype associated with a very dismal prognosis [4,5].

TNBC is more prevalent in premenopausal women younger than 40 years, African American women, and in persons who harbour the deleterious breast cancer susceptibility genes (BRCA 1 or 2) mutation [6]. Compared to other breast cancer subtypes, TNBC patients are often initially diagnosed at advanced stages and present with a more aggressive clinical course associated with a mortality rate of 40% within the first 5 years after diagnosis [5]. TNBC patients are prone to earlier recurrence with metastatic spread to distant organs including the lung, brain, liver, and bones compared to patients with other breast cancer subtypes. The mortality rate of TNBC patients within 3 months after recurrence is as high as 75% [7,8]. Frequently, pregnancy associated breast cancers display a higher incidence of the TNBC phenotype and have a poorer prognosis [3,5].

As TNBCs do not express ER, PR, or HER2, specific endocrine therapies (tamoxifen) or targeted therapies (trastuzumab) benefiting other breast cancer subtypes are ineffective in TNBCs. Therefore, in relation to other subtypes of breast cancer, TNBC has limited treatment options and is managed with conventional therapeutics such as surgery, radiotherapy, and chemotherapy, often leading to systemic relapse [9]. Given the lack of targetable receptors and the poor prognosis, it is critical to improve our understanding of TNBC at all levels to aid in the development of novel efficacious therapies.

There have been recent breakthrough therapies including poly ADP ribose polymerase (PARP) inhibitors olaparib and talazoparib in a subpopulation of TNBC patients who harbour germline BRCA1 or BRCA2 mutation [10,11]. Over the past few years, immune checkpoint inhibitor (ICI) drugs have started to make progress in the TNBC subtype with more promising outcomes [12]. Therefore, intensive research efforts have been focused on identifying predictive biomarkers for clinical response to ICIs and designing rational combination therapies of ICIs with other therapeutic agents that can synergize with ICIs to augment overall therapeutic efficacy. This review highlights how the interplay between immune checkpoint protein (ICP) expression, metabolic reprogramming (MR), and epithelial-to-mesenchymal transition (EMT) influences tumour growth and progression to metastasis of TNBC. Furthermore, we provide insights into potential therapeutic targets of metabolic vulnerabilities and cellular plasticity that can possibly synergize with ICIs to guide TNBC therapy in the future.

## 2. Metabolic Reprogramming in TNBC

During tumour progression, cancer cells are exposed to several metabolic stresses in their microenvironment such as changes in nutrients, varied oxygen levels and alterations in extracellular matrix molecules. To adapt to these metabolic challenges, cancer cells often rewire their metabolic state allowing for proliferation, invasion, and metastasis [13]. Metabolic reprogramming (MR) involving the rewiring of metabolic state is one of the major features of cancer and is recognized as a hallmark of cancer cells [14]. During MR, characteristics of metabolic enzymes, their modulators, and effector metabolic products, known as metabolites, are altered [15].

Otto Warburg was the first to identify MR in cancer cells. Named after him, the Warburg effect, also known as aerobic glycolysis, generates adenosine triphosphate (ATP) through increased levels of glucose uptake and lactate production, even in the presence of oxygen and fully functioning mitochondria. As a result, glycolytic intermediates required for biosynthetic processes are increased, while reactive oxygen species (ROS) from oxidative phosphorylation (OXPHOS) are reduced [16]. The Warburg effect leading to enhanced lactate production in turn induces an acidic microenvironment around the cancer cells, which enhances extracellular matrix remodelling, angiogenesis, and tumour invasion [17,18]. Following discovery of the Warburg effect, fatty acid metabolism (including fatty acid synthesis, oxidation, uptake, and storage) and amino acid metabolism have also been identified as key aspects of MR in cancers such as TNBC [19]. Strategies aimed at interventions targeting key vulnerabilities in MR may provide novel therapeutic approaches for TNBC and improvement of patients’ prognosis.

TNBC is characterised by discrete metabolic phenotypes that distinguish it from the other subtypes of breast cancer, leading to enhanced survival and proliferation under metabolic stress. For instance, from a study on relative metabolite quantification of 75 breast cancer patients without known distant metastases, glutamine expression is found to be reduced while choline and glycerophosphocholine (GPC) levels are increased in TNBC as compared to triple positive breast cancer [20,21]. This is suggestive of higher glutaminolysis, differential proliferation, and cell signalling in TNBC. Metabolomic profiling analysis of TNBC cell lines MDA-MB-231, MDA-MB-468, Hs578T, and HCC70 show dynamic alterations of glycolytic and OXPHOS rates and more heterogeneity as compared to other subtypes [22]. MDA-MB-231 and MDA-MB-468 have higher glycolytic activity and reduced OXPHOS than ER+ breast cancer cell lines [23]. Serine expression levels are higher in MDA-MB-231 and MDA-MB-468 as well as TNBC patient samples as compared to HER2+ cells [22]. The glucose transporter 1 (GLUT1) is also present in much higher levels in TNBC patient biopsies than other breast cancer subtypes [24]. Another study classified TNBCs into three heterogeneous metabolic-pathway-based subtypes with distinct metabolic features, namely, MPS1, the lipogenic subtype with upregulated lipid metabolism which was more susceptible to fatty acid synthesis inhibitors; MPS2, the glycolytic subtype with upregulated carbohydrate and nucleotide metabolism susceptible to glycolytic inhibition; and MPS3, the mixed subtype with partial dysregulation in the lipid, carbohydrate, and nucleotide metabolism pathways. Of interest, inhibition of lactate dehydrogenase sensitized these tumours to respond to anti-PD-1 immunotherapy in MPS2 TNBCs [Gong, Y. 2021] [25].

Several molecular features are responsible for such metabolic plasticity. The tumour suppressor p53 is mutated in 80% of TNBC, leading to increased proliferation within nutrient poor environments [26,27]. Higher losses of phosphatase and tensin homolog (PTEN), retinoblastoma (RB1), and phosphatidylinositol-4,5-bisphosphate 3-kinase catalytic subunit alpha (PIK3CA) expressions, along with beclin-1 (BECN1) mutation that causes MR by impairing autophagy, are particularly reported in TNBC [19]. The oncogene Myc, coding for C-myc, is amplified in 53% of TNBC [28]. C-Myc further facilitates tumour survival by reprograming critical metabolic pathway drivers such as hypoxia inducible factor 1-alpha (HIF-1 α) [29]. In the following sections, we summarize the current knowledge on the metabolic pathways involved in TNBC MR.

### 2.1. Glucose Metabolism and Glycolysis

Amongst all the breast cancer subtypes, extensive glycolysis from high glucose uptake is particularly essential for TNBC progression [30]. In normal non-cancerous cells, glucose is aerobically converted to pyruvate (through glycolysis) in the cytoplasm for transport to the mitochondria. The tricarboxylic acid cycle (TCA) and OXPHOS in mitochondria subsequently utilize the pyruvate for ATP generation [31]. In TNBC, the pyruvate produced in the cytoplasm is continually metabolized to lactate, preventing the mitochondrial uptake of pyruvate. Thus, lactate fermentation through increased aerobic glycolysis in the cytoplasm, rather than mitochondrial pyruvate metabolism, is the primary source of rapid energy production in the cancer cells [32]. A possible pathway for such enhanced aerobic glycolysis is linked to c-Myc based repression of thioredoxin-interacting protein (TXNIP); with high Myc and low TXNIP reported only in the TNBC subtype [33]. Moreover, glucose metabolism comprises of catabolic glycolysis for ATP generation, de novo serine and glycine synthesis, and the anabolic pentose phosphate pathway (PPP) for precursors of nucleotide synthesis. In a small scale study of biopsy samples from 12 TNBC patients, [1,2-13C] labelled glucose was provided as an infusion to differentiate between glycolysis and PPP, with results showing the predominance of glycolysis over PPP in TNBC [34].

The glycolytic intermediates are severely dysregulated in TNBC. Upregulation of HIF-1 α in response to the hypoxic tumour microenvironment causes significant overexpression of GLUT1 protein, responsible for glucose uptake across the cell membrane. Immunohistochemistry (IHC) and microarray studies from tissues of 132 TNBC patients revealed elevated intracellular glycolysis in 59.8% samples in conjunction with 65.2% GLUT1 overexpression in the tumour [30,35]. Serine/threonine kinase Akt and USP6 N-terminal like (USP6NL), glucose signal transduction regulators, are frequently upregulated in TNBC [36,37]. The PI3K/Akt is a key pathway in glucose homeostasis that is dysregulated in cancer, leading to aberrant cellular processes of proliferation, growth, metabolism, and metastasis. In a study including 97 TNBC and 36 non-TNBC tumour samples, IHC and formalin-fixed paraffin-embedded (FFPE) samples show 33% of TNBC have malfunctioning PI3K/Akt pathways; with overexpression of Akt and p-p44/42 MAPK proteins in all the samples bearing PIK3CA mutation [36,37]. Akt stabilizes GLUT1, and the crosstalk between the Akt signalling pathway and glucose metabolism is further enhanced by elevated levels of the GTPase-activating protein USP6NL. In TNBC cell lines HCC70 and HCC1187, reduced cell proliferation has been reported in aggressive TNBC upon targeted knockdown (KD) of overexpressed USP6NL [37].

The enzyme catalysing glucose phosphorylation, hexokinase 2 (HK2), transcriptional promoter activators miR-155 and miR-143, and pyruvate kinase 2 (PKM2) isoenzyme are further considered as potential targets for TNBC treatment [38,39,40]. In MDA-MB-231 cell line and mouse xenograft models, downregulation of HK2 reduced proliferation, increased apoptosis, and increased sensitivity to radiotherapy [38]. miRNAs play an extensive role in progression, proliferation, and metastasis in TNBC that impacts cancer cell metabolism. A thorough review is provided by Ding et al. on the miRNA landscape in TNBC [39]. In addition, miR-342-3p, which normally targets monocarboxylate transporter 1 (MCT1), is significantly downregulated in TNBC. This downregulation promotes the establishment of elevated glycolysis, and supply of exogenous miR-342-3p disrupts the glucose-lactate homeostasis in TNBC cell lines and tissues [41]. miR-210-3p, miR-105-5p, and miR-767-5p were recently revealed as glycolysis regulators in a large-scale screening of TNBC cell lines. miR-210-3p particularly promotes aerobic glycolysis in TNBC through the functional mediation of glycerol-3-phosphate dehydrogenase 1 like (GPD-1L; leading to HIF-1α stabilization) and cytoglobin (CYGB; supporting downregulation of p53) [42].

Normally, canonical OXPHOS of glucose intermediates occur in the mitochondria. Impairment of mitochondrial DNA plays a crucial role in the characteristic metabolic switching between elevated hypoxic glycolysis/lactate production and OXPHOS in TNBC. Interestingly, OXPHOS activity in TNBC is reported to be both increased as well as reduced [19,43]. In a study involving several breast cancer cell lines, it was found that activity of Complex III component of OXPHOS was absent, and Complex V was reduced by 90% in the triple-negative MDA-MB-231 [44]. The Complex I subunit is also found in lower levels in MDA-MB-231 but appears to be compensated by higher Complex IV activity [45]. On the other hand, RNA sequencing of 43 TNBC biopsy samples revealed significantly higher OXPHOS signature, with overexpression of Complex I genes that correlated with relapse and poor survival [46]. Moreover, the oncogenic deficiency of RB1, reported in 20–25% of TNBC derived cell lines, is proposed to facilitate OXPHOS and forms the therapeutic basis of the U.S. Food and Drug Administration (FDA) approved drug tigecycline for TNBC treatment [47,48]. The differential expressions observed can be partially explained based on a study involving TNBC cell lines as well as patient tissue samples. In the routinely used TNBC cell lines, mitochondrial metabolic reprogramming appears to be dissimilar to that seen in TNBC patient samples. Barely respiring MDA-MB-231 cells have three times lower mitochondrial levels that carry out 20% higher rates of pyruvate and glutamate conversion as compared to MCF-7. However, in samples from 59 breast cancer patients, the reverse was observed where TNBC possessed elevated mitochondrial activity as well as high respiration [49]. Table 1 provides an overview of some of the major components involved in MR of the glucose metabolism pathway in TNBC.

### 2.2. Amino Acid Metabolism

In addition to glucose, enrichment of amino acids is essential for tumour survival, proliferation, and invasion through EMT [52]. Glutamine, the most abundant circulating amino acid, is overexpressed in TNBC, along with higher levels of glutamine-related enzymes [53]. Glutamine catabolism acts as a carbon and nitrogen source and forms a precursor of glutathione. Indeed, metabolomics studies in TNBC have recorded low glutamine levels in conjunction with high glutamate levels, demonstrating elevated glutaminolysis [54]. The key regulators of glutamine metabolism and transport including glutaminase (GLS), the alanine-serine-cysteine-preferring transporter 2 (ASCT2 or SLC1A5), and L-type amino acid transporter 1 (LAT1 or SLC7A5) expression are enhanced in TNBC, causing mTORC1 nutrient sensing MR [54]. TNBC cell lines MDA-MB-231 and SUM-159PT and basal cell lines HCC38, HCC70, MDA-MB-468 and MDA-MB-157 were significantly dependent on glutaminase, with shRNA based glutaminase KD in MDA-MB-231 and SUM-159PT leading to carbon deprivation in TCA cycle metabolites and non-essential amino acids. A subsequent reduction in proliferation and significant apoptosis were also observed. In contrast, ER-positive (SKBR3) and luminal cell lines MDA-MB-453 and MCF7 were independent of glutaminase for growth and proliferation. Glutaminase KD with shRNAs of MDA-MB-231 and SUM-159PT as tumour xenografts in mice led to similar tumour regression in vivo [54]. The highest expressions of glutamine metabolism enzymes, however, is seen in HER2-positive subtype, and Luminal A tumours have the lowest reported levels [55].

While the reliance on glutamine has become a known metabolic feature of TNBC, results still remain heterogeneous within the subtypes. In a study on expression profiling of independently derived breast cancer cell lines covering all major subtypes, glutamine consumptions were higher in 11 TNBC, comprising 8 basal and 3 claudin low subtypes. Then again, the study found little to no significant glutamine elevation in 4 other claudin low TNBC derivatives; suggesting excessive nutrient intake needs not be mandatory for aggressive TNBC [56]. Pharmacological inhibition of glutaminase leads to reduced mammalian target of rapamycin (mTOR) expressions, and TNBC is especially sensitive to drug based mTORC1 suppression. Nevertheless, rather than exclusively targeting mTORC1 component of mTOR pathway, expression analysis profiling of patient-derived TNBC tumours in conjunction with in vivo studies on TNBC mice revealed a higher susceptibility of TNBC to mTOR targeting. The same study also found mTOR KD through RNA interference was more effective than targeting mTORC1 or mTORC2 separately [57].

Uptake of the amino acid cystine is necessary for maintenance of cancer stem cells in cancers including TNBC [58]. Luminal subtypes of breast cancer are reported to be cystine independent, whereas cystine deprivation has led to necrosis through mitochondrial fragmentation and ROS production in TNBC [19]. In as study where three human TNBC cell lines were cultured in different amino acid starvation media for 48 h, it was found that cystine starvation significantly induces necroptosis and ferroptosis in MDA-MB-231, Hs 578T, and HCC 1937. Interestingly, apoptosis or autophagy was not found to be involved in cystine-starvation-induced cell death in the TNBC cells [59]. In a similar study, 5 out of 6 basal-B cell lines (except MDA-MB-436) were found to be profoundly sensitive to cystine starvation, whereas most basal-A subtype cells and all luminal cell lines studied were not [60]. Of note, cystine deprivation MR seems to be especially correlated with the mesenchymal state and presents a characteristic feature of the basal subtypes seen in TNBC [60], representing a remarkable link with EMT that is further explored in Section 3.

Catabolism of the amino acids serine and glycine is needed for the synthesis of proteins, fats, nucleic acids, and co-factors. MR of the serine pathway, as compared to glycine, particularly appears to be essential for metastasis [61]. TNBC is characterised by elevated levels of serine and glycine metabolising enzymes, and their reduction decreases cancer cell proliferative capacity. Serine metabolism associated enzymes PHGDH and PSPH are reported to be highly expressed in TNBC cells MDA-MB-453S, MDA-MB-231, and MDA-MB-468. TMA of samples from 709 patients with invasive ductal carcinoma showed elevated expression of PHGDH, PSPH, and SHMT1 in the epithelial component of TNBC-type breast cancer. Clinicopathological analysis revealed tumour and stromal PSPH positivity and stromal SHMT1 negativity to be associated with short overall survival (OS) [62]. Asparagine, methionine, tryptophan, and arginine MR have also been reported to be necessary for impacting aggressive stem cells, EMT and apoptosis [63]. Several components of tryptophan catabolism, especially the kynurenine pathway and the aryl hydrocarbon receptor (AhR) are overexpressed in TNBC cell lines such as MDA-MB-231. The enzymes indoleamine 2,3-dioxygenase 1 (IDO1) and tryptophan 2,3-dioxygenase (IDO2 or TDO2) convert tryptophan to kynurenine, and their overexpression in TNBC cell lines promotes the metastasis of TNBC. Elevated TDO2 overexpression is also found to correlate with shorter OS in patient datasets [64,65]. Table 2 provides an overview of some of the major components involved in MR of amino acid metabolism pathways in TNBC.

### 2.3. Fatty Acid Metabolism

Along with glucose and amino acid metabolism, fatty acid or lipid metabolism is an integral component of the TNBC phenotype. Fatty acid synthesis (FAS) and its counterpart fatty acid oxidation (FAO) are efficient alternative energy sources for cancer cells [66]. FAO is vital for ATP, NADH, and NADPH production, required for survival, growth and metastasis in TNBC [66]. C-Myc overexpression in TNBC is linked with an increase in carnitine palmitoyltransferase (CPT) levels, a marker for increased reliance on FAO under hypoxic conditions [67].

In addition, FAS is reported to be both upregulated as well as down regulated in TNBC [19,43]. Normal non-cancerous breast cells utilize extracellular fats for biosynthesis. However, cancer cells increase de novo FAS to meet energy demands under metabolic stress [68]. One study looked at several TNBC cell lines (including developed doxorubicin-resistant TNBC cell lines) and 29 primary tumours and reported the overexpression of fatty acid synthase (FASN) in TNBC; with FASN inhibitors being used as adjuvants to achieve significant therapeutic benefits [69]. On the other hand, as compared to HER2 subtype of breast cancer, FAS and lipogenic enzyme levels have also been found to be reduced in TNBC while FAO appears to be enhanced and critically linked with MYC overexpression as seen from RNA-expression data from 771 breast cancer patients with primary human tumours [70]. These reports indicate the wide heterogeneity of the disease and the importance of further research to elucidate pathway details.

Triglycerides are a further source of fatty acids (FAs). CD36 is associated with the promotion and absorption of FAs by cells for cell growth. In TNBC, elevated levels of lipoprotein lipase (LPL) and CD36 expression have been reported [71,72]. LPL catalyses the hydrolysis of triglycerides into fatty acids, and CD36 is responsible for their cellular uptake. Cholesterol biosynthesis is also upregulated and essential for cancer stemness [73]. Phosphatidylcholine (PCPC) is the major phospholipid forming the eukaryotic cell membrane. In cancer cells, abnormal choline metabolism occurs through overexpression of choline kinase (Chk)-α, leading to aberrant PC synthesis [74]. Table 3 provides an overview of some of the major components involved in MR of fatty acid metabolism pathways in TNBC.

## 3. Synergy between Metabolic Reprogramming, Immune Checkpoints, and Epithelial-Mesenchymal Transition Lays the Foundation to TNBC Progression

**Figure 1:**
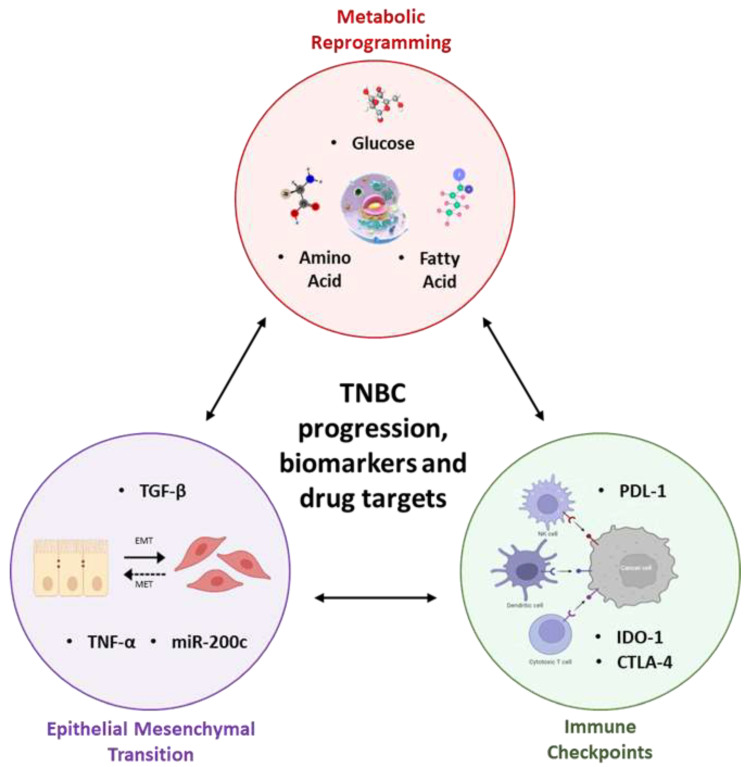
TNBC progression involves interplay of Metabolic Reprogramming, Immune Checkpoints, and Epithelial Mesenchymal Transition.

The interplay between MR, ICP levels, and EMT forms the cellular backdrop leading to the aggressiveness of TNBC and can be exploited to reveal key biomarkers and drug targets for therapy (Figure 1). MR sets up the stage for elevated ICP levels and immunosuppression in cancer, including TNBC. EMT is a developmental reprogramming event that is hijacked by tumour cells to potentiate aggressive properties of cancers including their metastatic dissemination [75].

Metabolic plasticity induces inactivation or improper functioning of the normal immune cells due to disruptions in glucose, lipid, or amino acid biosynthesis pathways [76]. Abnormally high glucose uptake (the Warburg effect) and the conversion of pyruvate to lactate in the cytoplasm lead to an acidic lactate rich environment, along with high levels of glucose transport and lactate associated enzymes. Such an environment, in turn, restricts cytotoxic CD8+ T cell lymphocytes (CTLs), natural killer (NK) cells, IFN-g, and interleukin (IL)-2 [77]. Elevated aerobic glycolysis in TNBC causes high secretion of granulocyte colony stimulating factor (G-CSF) and granulocyte-macrophage colony-stimulating factor (GM-CSF), further contributing to an immunosuppressive environment [78].

Moreover, high lactate increases PDL-1 levels leading to immune evasion through downregulation of T cell functions [77]. Increase in PDL-1 levels is also directly linked with glutamine deprivation, arginine and glutamate MR, and choline kinase-α (Chk-α) downregulation in abnormal choline metabolism [74,79,80]. Inhibition of the overexpressed PDL-1 has led to increased T cell memory function by impairing glycolysis and causing switch to FAO [81]. Anti-PD-1/PDL-1 therapy also increases sensitivity of tumour cells to CTLs and apoptosis [82]. Similarly, one study reported that inhibition of a key epigenetic regulator, lysine-specific demethylase 1 (LSD-1), enhances susceptibility of TNBC to ICP blocking antibodies such as anti PD-1 immunotherapy [83]. In another study on TNBC cell lines, Chk-α overexpression due to choline MR was found to be an important part of PDL-1 immunosuppression [74]. Interestingly, upregulation of Chk-α led to downregulation of PDL-1 and vice versa, indicating an inverse correlation between the two. Prostaglandin-endoperoxide synthase 2 (COX-2) and transforming growth factor beta (TGF-β) were shown to play a further part in the Chk-α—PDL-1 interactome [74].

Ca^2+^/Calmodulin (CaM)-dependent serine–threonine protein kinase (CaMKK2) has an intrinsic impact on cell cycle, cell differentiation, cytoskeletal structure, and hormone and cytokine production [84]. CaMKK2 also plays a crucial role in regulation of metabolic stress. One study reports high CaMKK2 expression in TNBC and shows CaMKK2 inhibition can significantly reduce tumour growth by increasing sensitivity to CTL mediated destruction [84]. Indoleamine 2,3-dioxygenase (IDO) is another important ICP, belonging to the tryptophan metabolism pathway [85]. IDO activity leads to immune evasion from regulatory T cells. A fascinating combination immunotherapy nanoplatform, based on IDO inhibition, has recently been shown to be highly promising for treatment in TNBC 4T1 model [85]. Over consumption of glutamine by the cancer cells, due to GLS overexpression in TNBC, is further reported to hamper tumour infiltrating lymphocytes (TILs) and aid immune evasion (Flores-Mendoza, 2021). One major function of CD8+ T cell destruction of targeted cancer cells is via Fas and Fas Ligand (Fas/FasL) interaction. Anti-PD-1/PD-L1 therapy renders tumour cells sensitive to CD8+ T cell and FasL-mediated lysis [Flores-Mendoza, 2021].

Upon activation of the developmental reprogramming event known as EMT, sessile epithelial cells typically shed the expression of epithelial markers, such as E-Cadherin, and transition to a mesenchymal cellular state with gain in expression of mesenchymal markers, such as N-Cadherin and Vimentin. These cells also acquire mesenchymal traits including invasiveness and motility, immune evasion, and ability to form tumour initiating cancer stem-cells (CSCs) and to acquire a heightened resistance to both chemotherapeutic and immunotherapeutic regimens [86,87]. The reverse process of EMT, termed mesenchymal to epithelial transition (MET) is another important feature of metastatic outgrowth [88].

We and others have reported that EMT has a key role in preventing the efficacy of immunotherapy [87,89,90]. Notably, the regulation of PD-L1 expression by EMT pathways to increase immunosuppression which exacerbates treatment resistance is well recognized [87,89,90]. EMT is both a consequence and a cause for MR that is essential for metastasis and TNBC progression. For instance, treatment of the mesenchymal MDA-MB-231 with EMT reverser miR-200c reduced cystine dependence; indicating cystine addiction to be a characteristic phenotype of mesenchymal state. Furthermore, TNFα seems to be almost consistently correlated with such cystine deprivation in basal subtypes and TNBC, with high TNFα activity appearing to be essential for cystine-deprived necrosis [66]. TNFα is a known EMT inducer in several tumours, and an increase in its activity largely leads to EMT progression. Prominent mesenchymal features are a common feature of TNBC. The enzymes IDO1 and IDO2 (or TDO2) convert tryptophan to kynurenine, and their increased levels promote immune evasion as well as EMT of TNBC. The kynureninase gene expression, functioning downstream to IDO and TDO, is also increased in TNBC. While predominance has so far been given to therapeutic targeting of IDO1, TNBC cell line studies revealed TDO2 as another strong therapeutic target and TDO2 overexpression is reported to correlate with shorter OS in patient datasets [64]. Indeed, results from plasma sample analysis show kynurenine directly mediates the reduced viability of CD8 T cells, leading to immunosuppression in TNBC, and this effect is overturned by TDO inhibitors [65].

Thus, metabolic vulnerabilities such as cystine deprivation and kynurenine pathway gene expressions represent EMT associations as viable targets for therapeutic exploitation in TNBC. Moreover, redundancies in the expression of multiple ICPs together with EMT may render cancer cells non-responsive to therapies targeting one or few checkpoints [89]. We and other have demonstrated that tumour cells with epithelial or mesenchymal status are differentially susceptible to immune attack [89,91,92].

## 4. Clinicopathological Overview

In a study on biopsy samples lacking any pre-surgical therapy of 75 breast cancer patients without known distant metastases, comparison of metabolite profiles between TNBC and TPBC revealed significantly higher choline levels in TNBC—indicating association of high choline levels with increase in tumour aggression [21]. This correlates with a metabolite study on pre- and post-treatment biopsies from 33 breast cancer patients where treatment comprised of tamoxifen administration for 5 years was followed by post-operative radiation therapy [93]. Patients with partial response, similar to patients with long-time survival, showed a trend of decrease in choline containing metabolites and significant reduction of glycerophosphocholine (GPC) post-treatment compared to patients with stable disease. In this report, GPC was found to be the best predictor of long-term survival and taurine as best predictor of treatment response. Conversely, non-survivors had no significant changes in choline-containing metabolites (tCho) post-treatment [93]. Similarly, another report in breast cancer patients found neoadjuvant chemotherapy responders to show decreased tCho levels at early stage (24 h) as well as following treatment completion as compared to non-responders [94].

Based on Glut1 and CAIX expression in tumour and stroma of tissue samples from 132 breast cancer patients, TNBC was metabolically divided into Warburg type (most common, 59.8%), reverse Warburg type (least common, 5.3%), mixed metabolic type (18.2%), and metabolic null type (16.7%) [35]. Glycolysis in tumour and non-glycolytic stroma was considered as Warburg type, non-glycolytic tumour and glycolysis in stroma as reverse Warburg type, glycolysis in both tumour and stroma as mixed metabolic type, and non-glycolytic tumour as well as stroma as metabolic null type. Correlation with clinicopathological factors revealed high tumoral MCT4 expression in basal-likes as compared to metabolic null types and association of Warburg type TNBC with younger age. While shorter OS was associated with negative stromal MCT4 expression, the authors report this in contrast to other reports where positive stromal MCT4 expression was found to be predictive of decreased OS, citing methodological differences as the probable cause of differential observations [95]. However, a point of note is that no significant differences were seen in the metabolic phenotypes within the molecular subtypes of TNBC [35].

The ICI PDL-1 as well as LDHA are linked with worse OS and DFS [96]. PDL-1 was associated with worse OS and DFS in TNBC. In addition, PDL-1 had prognostic significance with more than 50% TNBC patients having positive PDL-1 expression. LDHA also displayed a similar pattern with worse OS and DFS in TNBC tissues. In a likewise observation, positive expression of both PDL-1 and LDHA correlated with shorter OS and DFS [96]. In a related study on clinicopathological analysis of samples from 112 breast cancer patients, while statistical significance could not be observed, tissue microarray revealed TNBC patients possessed positive expression of both LDHA and AMPK as compared to non-TNBC patients [97]. LDHB is also linked with poor OS in aggressive breast cancers such as TNBC. In a study that analysed outcome data in a cohort of 227 patients, high LDHB expression correlated with both poor OS and progression-free survival, and this association was found for both basal and luminal subtypes [98]. Furthermore, a recent thorough analysis of the proteomic profile in 88 TNBC cases revealed the immune features of four TNBC subgroups with disparate survival outcomes. Most favourable survival correlated with the cluster enriched for immune-related pathways such as type I and type II IFN signalling, MHC class I subunits, and other antigen presentation biomarkers [99]. Such studies provide an insight on immune checkpoint inhibition therapies other than typical PD-1/PDL-1 clinical tests and represent avenues for improved or adjuvant biomarkers or therapeutic candidates.

When it comes to the mitochondrial metabolism marker MCT1, responsible for lactic acid transport and regulation of extracellular tumour pH, TNBC is significantly associated with elevated MCT1 expression. IHC of 249 FFPE breast cancers revealed a significant increase in MCT1 expression as compared with normal tissues and correlated with basal-like subtypes, high histological grade, negative ER and PR expression, CK5 and CK14 expression, and Ki67 expression [100]. Out of 31 TNBC samples, 26% stained with high MCT1 expression [98]. In a more detailed study including 532 human samples of invasive breast cancer, derived from 257 individual patients, a higher high MCT1 expression correlated with TNBC as compared to the other subtypes with an evaluated difference of 27%. Higher MCT1 expression displayed a link with OS, but this was not statistically significant. However, MCT1 was a significant predictor of recurrence [50].

OXPHOS is a canonical pathway that is dysregulated and correlates with increase in tumour aggressiveness and TNBC. RNA sequencing analysis of pre-treatment biopsies from 43 operable TNBC patients treated with taxane and anthracycline-based neoadjuvant chemotherapy showed higher risk of relapse and lower risk of survival associated with elevated expression of OXPHOS signature, especially Complex I. On generating patient derived xenografts (PDXs) from the treated patient samples, targeting OXPHOS with IACS-10759 (a novel Complex I inhibitor currently in clinical trials) resulted in wide antitumour efficacy for TNBC growth, with highest inhibition seen in basal-like TNBC [46].

Glutaminolysis inhibitors also recently reached clinical trials in TNBC. However, a clear correlation between glutaminase inhibition in TNBC clinical samples was found only at a hypothetical stage, possibly due to low sample numbers (6 TNBC patients out of 59 human breast cancer samples). In fatty acid metabolism, both FAO and FAS expressions have been evaluated for clinical intervention and therapeutic potential. FASN inhibition including combination treatment with doxorubicin, C75, cetuximab and EGCG showed a strong synergistic effect in chemo-sensitive and chemo-resistant TNBC cell lines, biopsy samples of 29 TNBC patients and orthoxenograft mice models [69]. Analysis of The Cancer Genome Atlas (TCGA) breast cancer dataset including 771 patients found reduced ACC2 gene expression, indicative of increased FAO, to correlate with the worse prognosis and outcome in the TNBC cohort [70]. Cholesterol biosynthesis-associated proteins have been found to be elevated in TNBC cell lines, and a cohort of 615 basal-like breast cancer patients revealed a significant correlation with high gene expression and shorter relapse-free survival [73].

## 5. Future Direction

Elucidating the mechanisms and interplay between ICs, EMT, and MR will be essential in developing novel reliable biomarkers and drug targets for TNBC patients. A number of techniques like MR spectroscopy, LC-MS/MS, and GC-MS have been used to address MR in TNBC, but extending these studies to include cellular plasticity will be important. Combinatorial techniques like immunohistochemistry followed by Matrix assisted laser desorption ionisation–mass spectrometry imaging (MALDI-MSI) to visualise spatial distribution of lipids and metabolites in TNBC patient biospecimens would provide region-specific distribution profiles. This could be coupled with laser capture microdissection (LMD) to cut out specific regions of interest (ROI) from the tissues for proteomics profiling with LC-MS/MS [101]. Mapping the profiling data to the ROIs can provide vital information about the tumour and TME. Major advances in mass spectrometry hardware now enable deep profiling capabilities at the single-cell level [102,103]. Use of artificial intelligence to integrate high-resolution imaging data to ‘omics’ datasets (including transcriptomics) would provide an ideal function-based snapshot of the biospecimen. Furthermore, incorporating spatially resolved technologies such as digital spatial profiling will allow the study of EMT components, immune checkpoints, and MR pathways in relation to each other and will reveal more complex mechanisms underlying MR, EMT, and ICs in cancer cells. While the initial results with immune checkpoint therapies in TNBC patients are encouraging, it remains to be determined which therapeutic regimen targeting immune checkpoints will ultimately have the greatest impact on improving TNBC patient outcomes: in conjunction with EMT pathway inhibitors and/or metabolic adaptation targeting drugs; or used in combination with drugs that target both cellular plasticity and MR.

## Figures and Tables

**Table 1:** Glucose metabolism and glycolysis in TNBC.

Gene/Protein/Pathway	Status in TNBC	Study Model	Clinicopathological Evaluation	Reference
Oxidative phosphorylation	Glycolysis dominates over oxidative phosphorylation	12 TNBC patients, 12–14-week-old male C57BL/6 mice	Not evaluated	[34]
	Complex III component absent and Complex V was reduced by 90% in MDA-MB-231	several breast cancer cell lines	Not evaluated	[44]
	Lower levels of Complex I subunit, higher levels of Complex IV in MDA-MB31	Cancer cell lines MCF-7, MDA-MB-231, T47D, and MDA-MB-435	Not evaluated	[45]
	High OXPHOS signature, with overexpression of Complex I	43 TNBC biopsy samples	Correlation with relapse and poor survival	[46]
GLUT1	65.2% overexpression	132 TNBC patients	Not evaluated	[30,35]
PI3K/Akt pathways	overexpression of Akt and p-p44/42 MAPK proteins in samples bearing PIK3CA mutation; elevated GTPase-activating protein USP6NL	97 TNBC and 36 non-TNBC tumour samples; TNBC cell lines HCC70 and HCC1187, human mammary epithelial cell line HMEC and non-small cell lung carcinoma cell line H1299	Not evaluated	[37,38]
Hexokinase 2 (HK2)	HK2 suppression improves radiosensitivity	TNBC cell line MDA-MB-231; 6-week-old female BALB/c mice with MDA-MB-231 injections.	Not evaluated	[38]
LDHA and PDL-1	PDL-1 elevated in TNBC cell lines (especially HCC38 and MDA-MB-231 cells) and in 17 of 20 patients. LDHA elevated in TNBC cell lines compared to MCF-10A and in 18 of 20 patients.	human mammary epithelial (HME) cell line MCF-10A and TNBC cell lines MDA-MB-453, MDA-MB-468, MDA-MB-231, BT-549, HCC38 and 4 T1; 20 TNBC tissues and normal adjacent tissues; tissue microarrays (TMAs) of 554 cases of breast cancer tissues	PDL-1 and LDHA are linked with worse OS and DFS	[50]
MCT1	Elevated expression in TNBC as compared with ER+ and/or PR+ and HER-2+ (*p* < 0.001).	TMAs of 257 cases of breast cancer tissues	Increased MCT1 predictive of recurrence. However, correlation with OS is not significant.	[50]
MCT4 and CD147	Enhanced expression; correlating with poor prognosis and metastases	249 FFPE breast cancer samples	Not evaluated	[51]

**Table 2:** Amino acid metabolism in TNBC.

Gene/Protein/Pathway	Status in TNBC	Study Model	Clinicopathological Evaluation	Ref.
Glutaminase, ASCT2 or SLC1A5, LAT1 or SLC7A5 (all up)	Low glutamine levels in conjunction with high glutamate levels, demonstrating elevated glutaminolysis	TNBC cell lines MDA-MB-231 and SUM-159PT, and basal cell lines HCC38, HCC70, MDA-MB-468 and MDA-MB-157; ER-positive (SKBR3) and luminal cell lines MDA-MB-453 and MCF7	Not evaluated	[54,55]
Glutamine sensitivity	Out of cell lines analysed, 11 TNBC (3 basal, 8 claudin low) identified as ‘glutamine auxotrophs’.	Metabolic profiles of 46 independently-derived breast cell lines	Not evaluated	[56]
RTK signalling pathways, mTOR activation	Most frequently activated: EGFR, 75% of patients, HER2 33% of patients, HER4 25% of patients, PDGFRβ; 71% of patients, Akt (88% of patients), Erk1/2; 53% of patients.	26 patient derived TNBC tumours; HBL100, HS578T and MDA-MB-231 cell lines; TNBC mice	In vivo analysis of BEZ235 combinations with taxotere or carboplatin—delay in tumour growth observed.	[57]
Cystine susceptibility	Most essential amino acid for TNBC cell growth—Starvation led to necroptosis and ferroptosis in MDA-MB-231, Hs 578T, and HCC 1937. Positively correlates with CHAC1 expression.	Cell lines: MDA-MB-231, Hs 578T, HCC 1937, MCF-7	Not evaluated	[59]
	EMT strongly correlates with cystine dependence.	luminal (BT474, ZR751, MCF7) and basal (MDA-MB-157, BT20, MDA-MB-231) subtypes	Not evaluated	[60]
Serine/glycine metabolising enzymes	High expression of PHGDH and PSPH in TNBC cell lines; elevated PHGDH, PSPH, and SHMT1 in epithelial component of TNBC cancer.	Cell lines: MCF-7, MDA-MB-361, MDA-MB-453, MDA-MB-435S, MDA-MB-231, and MDA-MB-468; tissue samples from 709 patients with invasive ductal carcinoma	Not evaluated	[62]
Tryptophan catabolism and AhR signalling	Tryptophan 2,3-dioxygenase (TDO2), kynureninase (KYNU) upregulated in suspension as compared to attached cultures of SUM159PT, MDA-MB-231, BT549. TDO2 inhibition decreased metastasis in vivo.	Cell lines: SUM159PT, MDA-MB-231, BT549, MCF7 and T47D. Tail-vein injection of MDA-MB-231 cells into NOD.CB17-Prkdcscid/J (NOD/SCID) mice. primary TNBC patient samples. Gene expression microarray from Curtis et al. dataset (*n* = 1998).	Above-median TDO2 expression had about 3 years shorter OS as compared with below-median. expression (*p* = 0.0002). Above-median IDO1 expression had a shorter OS than with reduced IDO1, but difference was less than TDO2 expression.	[64]
Kynurenine, tryptophan 2,3-dioxygenase (TDO)	Induces CD8 T cell death and reduces its viability.	Cell lines: BT549 cells. Pre-surgical breast cancer patient plasma (*n* = 77) and tumour-free donors (*n* = 40).	Not evaluated	[65]

**Table 3:** Fatty acid metabolism in TNBC.

Gene/Protein/Pathway	Status in TNBC	Study Model	Clinicopathological Evaluation	Ref.
FAS and FASN enzyme	Expression of FASN in TNBC cell lines, sensitivity to FASN inhibitors especially in doxorubicin resistant models.	TNBC cell lines MDA-MB-231, MDA-MB-468, MDA-MB-157, HCC1806, Du4475, and BT549, Doxorubicin-resistant cells MDA-MB-231 (231DXR) and HCC1806 (HCCDXR); paraffin-embedded biopsy samples of 29 TNBC patients; orthoxenografts of NRG (NOD-Rag1<null> IL2rg<null>) mice.	Combination treatment with doxorubicin, C75, cetuximab and epigallocatechin gallate (EGCG) showed a strong synergistic effect in chemo-sensitive and chemo-resistant cells.	[69]
MYC-dependent FAO dysregulation	FAO upregulated in TNBC mice model that correlates with high MYC expression, FAS is preferntial through ACC1 activity rather than ACC2, treatment with etomoxir in cells with high MYC reduced proliferation but not viability.	MMTV-rtTA/TetO-MYC mice, orthotopic allograft and xenograft in NOD/SCID female mice and WT FVB/N models; The Cancer Genome Atlas (TCGA) breast cancer dataset (771 patients); cell lines—human mammary epithelial cell line HMEC, six TN and three RP cell lines HCC1428 and T47D	Reduced ACC2, a critical FAS gene, indicative of increased FAO that leads to aggressiveness of breast tumours, is linked with worse prognosis and outcome in TNBC cohort.	[70]
Lipoprotein lipase (LPL) and CD36 overexpression	TNBC cells Du4475 expressed the highest levels of LPL mRNA and lowest levels of heparanase mRNA.	45 human breast cancer cell lines (ICBP45)	Not evaluated	[71]
CD36 overexpression	Lower dose of genistein combined with CD36 siRNA loaded nanoparticles suppressed proliferation and promoted apoptosis.	Cell lines: MDA-MB-231	Not evaluated	[72]
Cholesterol biosynthesis	Increase in expression of cholesterol biosynthesis associated proteins.	Cell lines MDA-MB-468 and MDA-MB-231; cohort of 615 basal-like breast cancer patients	Cholesterol biosynthesis-associated proteins significantly correlated with high gene expression and shorter relapse-free survival in the basal-like cohort.	[73]
Chk-α and PD-L1	High expression and interdependence of Chk-α and PD-L1	Cell lines MDA-MB-231, SUM-149; TCGA TARGET GTEx database	Not evaluated	[74]
